# Identification of Novel Short C-Terminal Transcripts of Human *SERPINA1* Gene

**DOI:** 10.1371/journal.pone.0170533

**Published:** 2017-01-20

**Authors:** Nerea Matamala, Nupur Aggarwal, Paolo Iadarola, Marco Fumagalli, Gema Gomez-Mariano, Beatriz Lara, Maria Teresa Martinez, Isabel Cuesta, Jan Stolk, Sabina Janciauskiene, Beatriz Martinez-Delgado

**Affiliations:** 1 Molecular Genetics Unit, Instituto de Investigación de Enfermedades Raras (IIER), Instituto de Salud Carlos III (ISCIII), Madrid, Spain; 2 Department of Respiratory Medicine, Hannover Medical School, Hannover, Biomedical Research in Endstage and Obstructive Lung Disease Hannover (BREATH), Hannover, Germany; 3 Department of Biology and Biotechnologies, Biochemistry Unit, University of Pavia, Pavia, Italy; 4 Respiratory Medicine Department, Coventry and Warwickshire University Hospital, Coventry, Warwickshire, United Kingdom; 5 Pneumology Department, Hospital 12 de Octubre, Madrid, Spain; 6 Bioinformatics Unit, Instituto de Salud Carlos III (ISCIII), Madrid, Spain; 7 Department of Pulmonology, Leiden University Medical Center, Leiden, Netherlands; Medizinische Fakultat der RWTH Aachen, GERMANY

## Abstract

Human *SERPINA1* gene is located on chromosome 14q31-32.3 and is organized into three (IA, IB, and IC) non-coding and four (II, III, IV, V) coding exons. This gene produces α1-antitrypsin (A1AT), a prototypical member of the serpin superfamily of proteins. We demonstrate that human peripheral blood leukocytes express not only a product corresponding to the transcript coding for the full-length A1AT protein but also two short transcripts (ST1C4 and ST1C5) of A1AT. In silico sequence analysis revealed that the last exon of the short transcripts contains an Open Reading Frame (ORF) and thus putatively can produce peptides. We found ST1C4 expression across different human tissues whereas ST1C5 was mainly restricted to leukocytes, specifically neutrophils. A high up-regulation (10-fold) of short transcripts was observed in isolated human blood neutrophils after activation with lipopolysaccharide. Parallel analyses by liquid chromatography-mass spectrometry identified peptides corresponding to C-terminal region of A1AT in supernatants of activated but not naïve neutrophils. Herein we report for the first time a tissue specific expression and regulation of short transcripts of *SERPINA1* gene, and the presence of C-terminal peptides in supernatants from activated neutrophils, *in vitro*. This gives a novel insight into the studies on the transcription of *SERPINA1* gene.

## Introduction

Gene expression is responsible for the synthesis of functional gene products, typically proteins. The question of how many genes are present in the human genome led to the collection of known protein-coding genes not only in the human genome but also in *Arabidopsis*, worm, fly and mouse [1–4]. The findings revealed that there is a significantly greater amount of transcriptional output from genomes than anticipated by the collection of annotated protein-coding transcripts [5]. New proteomic high-throughput technologies provide a strong evidence of the existence of non-canonical protein isoforms generated by the translation outside of the annotated protein-coding genes [6,7]. A variety of short open reading frames (ORFs), long noncoding RNAs and pseudogenes are coding short transcripts [8,9], which may express unrecognized biological functions.

The serpins (serine proteinase inhibitors) are proteins found in plants, animals, and viruses [10–12]. These proteins participate in the regulation of the coagulation, fibrinolysis, complement activation, angiogenesis, apoptosis, inflammation, neoplasia, viral pathogenesis, among others [13]. Human genome encodes 16 serpin clades, termed A to P. So far, 36 human serpins have been identified, among which 29 have a primary function in the regulation of the proteolytic activity. Mutations in serpin genes are associated with different human diseases, globally named serpinopathies [14]. Alternative splicing results in multiple transcript variants of serpin genes, however, to our knowledge; current databases contain no information about noncoding transcripts in human serpins.

Human *SERPINA1* gene is located on chromosome 14q31-32.3 [15], which covers approximately 12.2 kb, and has four coding exons, three untranslated exons and six introns. *SERPINA1* gene is expressed and translated in different tissues, and encodes alpha1-antitrypsin (A1AT), a prototypical member of the serpin superfamily of proteins. The regulation of A1AT expression is controlled by different promoters and transcription start sites located in the 5’UTR of the gene [16–18]. In hepatocytes, transcription typically begins in exon 1C [17,19] whereas in monocytes and macrophages A1AT transcribed mainly from exons 1A and 1B [16,18,20]. One particular feature of SERPINA1 gene is its high transcriptional complexity i.e. according to the Emsembl Genome Browser, human *SERPINA1* gene has 19 transcripts (splice variants), generated in a stimulus- and cell-type specific manner [21–23]. Different types of alternative splicing mechanisms generate this complexity of transcripts, including exon skipping and alternative 5’ and 3’ splice site usage. Most of the *SERPINA1* transcript variants are produced by the alternative splicing between the non-coding exons 1A, 1B and 1C.

Remarkably, a recent study on ovine *SERPINA*1 gene expression found short transcripts lacking exons II or III [24]. Because the *SERPINA1* gene exon and intron organization is similar among the species, theoretically short transcripts can also be present in human *SERPINA1* gene. In support of our prediction, herein we identified novel short transcripts of *SERPINA1* gene and provided evidence for tissue specific expression and regulation of these transcripts.

## Materials and Methods

### RNA extraction, cDNA synthesis and *SERPINA1* expression analysis by RT-PCR

Peripheral blood samples were obtained from subjects previously tested for A1ATD, and informed consent for this study was signed. The study was approved by the ethics committee of Instituto de Salud Carlos III. RNA isolation from peripheral blood leukocytes was performed using RNAeasy kit (Quiagen) following manufacturer’s recommendations. The cDNA synthesis was generated using the Maxima First Strand cDNA Synthesis kit (Thermo Scientific). To amplify *SERPINA1* transcripts, primers located in exon 1C: 1C_F:5’ctgtctcctcagcttcaggc3’ and 1A: 1A_F:5’tgaggagagcaggaaaggaca3’ were used in combination with a reverse primer located in exon V, EX5_R:5’ccatgaagaggggagacttgg3’. The PCR was performed under the following conditions: 35 cycles of 94°C for 45s, 60°C for 45s, and 72°C for 45s. Amplified products were visualized in 1–2% agarose gels. PCR products were purified by using PCR purification Kit (Qiagen) and subsequently cloned into pGEM-T easy vector (Promega). Ligation reactions were used to transform DH5α competent cells. Clones containing PCR products were selected by blue/white colony screening and standard ampicillin selection. Positive transformants were analyzed by the PCR and sequenced using *SERPINA1* primers.

### Quantification of ST1C4 and ST1C5 by QT-PCR

The expression levels of two short transcripts, ST1C4 and ST1C5, were independently analyzed by real time quantitative PCR (qPCR). cDNA samples were diluted and amplified using Taqman Fast advance master mix in a 7500 Fast Real Time PCR System (Applied Biosystems) with recommended PCR cycle conditions. To amplify the ST1C4 or ST1C5 transcripts two different reactions were performed using boundary-spanning primers for the sequence covering the 1C-exon4 junction ST1C4_F:5’tgggacagtgaatc/gtctgc3’ or the spanning region between 1C-exon5, ST1C5v3_F:5’acctgggacagtgaatc/gccgtg3’ in combination with a reverse primer in exon 5 for both reactions EX5_R:5’ccatgaagaggggagacttgg3’. Specific fluorescent-labelled Taqman probes were selected from the Universal probe library, UPL, Roche. UPL probe #57 was used for the ST1C4 transcript and the probe #3 was selected for the ST1C5 transcript. GUSB gene was used as an endogenous control to normalize the expression levels of the transcripts. All experiments were performed in triplicate and data were analyzed by Applied Biosystems 7500 software v2.0. Relative expression was calculated by using the comparative Ct method and obtaining the fold-change value (ΔΔCt).

We also analyzed *SERPINA1* full transcripts and compared these with expression of identified short transcripts. We individually analyzed alternative full transcripts generated by the use of exons 1A, 1B and 1C by using primers and methods reported before [25].

### ST1C4 and ST1C5 expression in cDNA samples from human tissues

We used a set of first-strand cDNAs from different tissues for analysis of short transcripts ST1C4 and ST1C5. We analyzed 16 commercially available first-strand cDNAs from 16 different human tissues (Human MTC Panel I and II, Clontech) as described before [25]. We performed at least two experiments in triplicate for all tissues. We normalized the expression of short transcript to the expression obtained in a leukocyte sample.

### PBMCs and neutrophil isolation from human blood

Human Peripheral blood mononuclear cells (PBMCs) were isolated from blood of healthy volunteers using Lymphosep discontinuous gradient centrifugation. PBMCs were re-suspended at a density of 5 x 10^6^ cells/ml in RPMI-1640 with 2 mM N-acetyl-L-alanyl-L-glutamine (Gibco, Life Technologies) supplemented with 1% nonessential amino acids, 2% sodium pyruvate and 20 mM HEPES and plated. We incubated cells for 75 min at 37°C and 5% CO_2_ to allow monocytes to adhere to the cell culture plates. Afterwards, we removed non-adherent cells by washing with PBS containing Mg2+ and Ca2+ (Gibco, Life Technologies) and fresh medium was added. Next day, adherent PBMCs were used for experiments.

Neutrophils were isolated from the freshly obtained peripheral blood of healthy donors using PolymorphoprepTM (Axis-Shield PoC AS, Oslo, Norway) according to the manufacturers’ recommendations. The neutrophil purity was typically 98% as determined by cytospins and cell viability was more than 90% according to trypan blue staining. Before starting the experiment, neutrophils (3x10^6^ per well) were always left alone for 30–40 min in cell culture plates pre-coated with fetal calf serum.

Freshly isolated neutrophils were treated with 10 ng/ml LPS for 5h. In parallel, cells were incubated with appropriate buffer.

### Sample preparation and LC-MS analysis

C-10, C-21 and C-36 standard peptides (JPT, Innovative peptide solutions, Berlin, Germany) were dissolved in RPMI Medium, to obtain a final concentration of 20 μM. All supernatants from neutrophil cultures (controls and LPS-stimulated) were filtered using Microcon^®^ centrifugal filters (Millipore Corporation, Bedford, MA, USA) with 10 kDa molecular weight cut-off (MWCO), to remove any large protein and concentrate the C-terminal peptides. 100 μL aliquots of both standard solutions and real samples were acidified by addition of 2 μL of formic acid (FA), to reach a final pH of about 3.0 was reached, and analyzed by LC-MS.

The LC-MS system (Thermo Finnigan, San Jose, CA, USA) consisted of a thermostated column oven Surveyor autosampler controlled at 25°C; a quaternary gradient Surveyor MS pump; a diode array detector and a Linear Trap Quadrupole (LTQ) mass spectrometer with electrospray ionization ion source controlled by Xcalibur software 2.0.7. Analytes were separated by RP-HPLC on a Jupiter (Phenomenex, Torrance, CA, USA) C_18_ column (150 x 2 mm, 4 μm, 90 Å particle size) using a linear gradient (2–60% solvent B in 60 min) in which solvent A consisted of 0.1% aqueous FA and solvent B of acetonitrile (ACN) containing 0.1% FA. Flow-rate was 0.2 mL/min. Mass spectra were generated in positive ion mode under constant instrumental conditions: source voltage 5.0 kV, capillary voltage 46 V, sheath gas flow 40 (arbitrary units), auxiliary gas flow 10 (arbitrary units), sweep gas flow 1 (arbitrary units), capillary temperature 200°C, tube lens voltage –105 V. MS/MS spectra, obtained by CID studies in the linear ion trap, were performed with an isolation width of 3 Th *m/z*, the activation amplitude was 35% of ejection RF amplitude that corresponds to 1.58 V. All analyses were performed in triplicate.

### Statistical analysis

Statistical Package (SPSS for Windows, release 21.0) was used for the calculations. An independent two-sample t-test was used. The differences in the means of cell experimental results were analyzed for their statistical significance using one-way ANOVA combined with a multiple-comparison procedure (Scheffe multiple range test), with an overall significance level of p = 0.05.

## Results and Discussion

### Identification of *SERPINA1* short transcripts

Peripheral blood leukocytes (e.g., T cells, monocytes, neutrophils) are readily accessible source of information about the health and physiological state of an individual. By comparing gene expression profiles of leukocytes, it is possible to identify genes with cell-type-specific expression patterns [26]. To analyze *SERPINA1* expression we prepared cDNA from human peripheral blood leukocytes and used a primer pair for a full-length *SERPINA1* cDNA (1C_F and EX5_R) to amplify SERPINA1 transcripts. When agarose gels visualized amplified products, we identified an expected 1279 bp band corresponding to the full-length A1AT. Cloning and sequence analyses confirmed the presence of the exon 1C and four coding exons (exon II to exon V) encoding the 419 aa A1AT protein. This transcript corresponded to the Ensembl transcript ENST00000393087.

**Fig 1:**
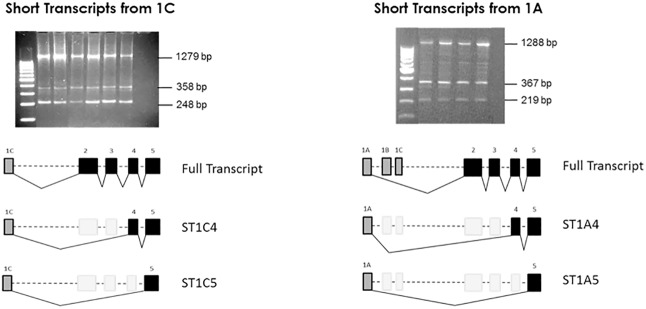
Expression of A1AT transcripts in peripheral blood cells. Gel electrophoresis shows cDNA transcript variants of SERPINA1 gene generated from exon 1C or exon 1A. Three different transcripts were obtained by PCR using RNA extracted from whole blood samples. The larger bands in both cases corresponded to the full length SERPINA1 transcript. The other lower bands represent short transcripts of the gene which included the untranslated exons IC or IA joined to exon IV and V (ST1C4 and ST1A4) or joined to exon V (ST1C5 and ST1A5).

Surprisingly, in addition to the 1279 bp band, we constantly observed two bands of few hundred base pairs, one of which (a band of 358 bp) revealed a short transcript of A1AT (Fig 1). After cloning, several colonies were obtained and sequencing showed a transcript with exon IC directly spliced to exon IV (ST1C4), with an exon structure IC-IV-V, lacking exons II and III. A shortest band of 248 bp revealed another transcript of A1AT with exon IC joined to exon V (ST1C5) missing exons II, III and IV. Sequence information of these new short transcripts is available through the following NCBI GenBank accession numbers: KU535895 (ST1C4) and KU535894 (ST1C5).

Since SERPINA1 alternative splicing events typically occur among the first non-coding exons, we investigated whether the newly identified short transcripts can possibly originate from exon IA. Using a primer pair located in exon IA (1A_F) and exon V (EX5_R), three main bands and few less evident bands occurred, similarly as we found by using the primer at exon IC (1C_F) (Fig 1). The band of 1288 bp represented the full SERPINA1 transcript starting at exon IA joined to the rest of coding exons II, III, IV and V. This transcript corresponded to the Ensembl transcript ENST00000355814.

Several colonies expressing an intermediate band of 367 bp were obtained, and sequencing analysis revealed a transcript structure containing exon IA directly bound to exons IV and V (ST1A4) (Fig 1). Finally, a shortest band of 219 bp was also cloned and sequencing analysis revealed a short transcript with exon IA directly joined to exon V, lacking all other coding exons (ST1A5). Sequence information of SERPINA1 short transcripts transcribed from exon IA can be accessed through NCBI GenBank accession numbers: KU755451 for the ST1A4 short transcript and KU755450 for the ST1A5 short transcript. Altogether, our results demonstrate that short transcripts can be transcribed from either exon IC or IA.

A previous study on ovine *SERPINA1* gene expression also found short transcripts lacking exons II or III [24]. However, human genomic databases (Ensembl or UCSC genome browser) do not contain information regarding short transcripts of *SERPINA1* gene identified in this study. Importantly, however, we found a RNA-Seq data set [27], presenting expression and alternative splicing analysis of blood leukocytes derived from controls and Parkinson's patients. In this data set, we found evidence supporting the presence of SERPINA1 short transcripts. By looking at single reads that map to different exon/exon junctions, we identified several reads, in which the exon IA binds directly to exon IV, lacking the exons II and III, similar as occurred with the transcript ST1A4 described here.

**Fig 2:**
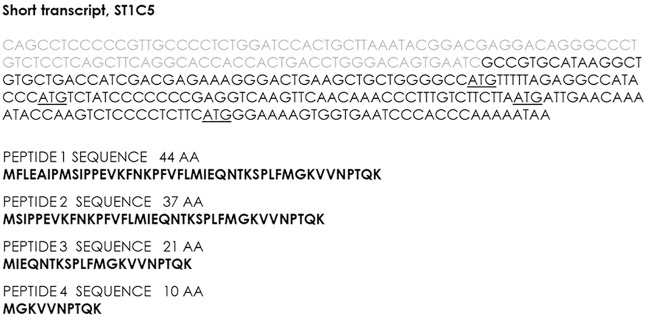
Sequence of the short transcript ST1C5. The exon 5 sequence has an ORF with several in frame ATG codons (these are underlined). Predicted amino acid sequences of potential peptides of 44, 36, 21 and 10 amino acids, generated by these ORF are shown.

Interestingly, in silico analysis showed that, ST1C4 and ST1C5 as well as ST1A4 and ST1A5 have an ORF in exon V starting at Met351 of the full-length A1AT protein. Further analysis confirmed that in the exon V, in addition to Met351, there are three more methionine residues. Hence, putative peptides generated by the short transcripts would have the same C-terminal amino acid sequence as a full-length A1AT protein, i.e. the last 44aa, 36aa, 21aa or 10aa, respectively (Fig 2).

### Differential expression and induction of short ST1C4 and ST1C5 transcripts in blood monocytes and neutrophils by LPS

**Fig 3:**
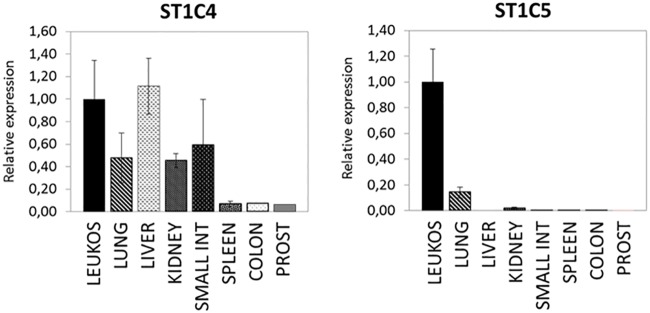
Expression profiles in different tissue samples. Tissue expression of short transcripts ST1C4 and ST1C5. Short transcripts show differential expression patterns in human tissues. Data were normalized to the expression detected in leukocytes. Histograms represent mean expression values of two experiments performed in triplicates. Bars represent mean (SD). LEUKO: Leukocytes, SMALL INT: Small Intestine, PROST: Prostate.

To investigate, if short transcripts of *SERPINA1* gene have a tissue specific expression, we performed a quantitative analysis focusing on ST1C4 and ST1C5, using cDNA from various human tissues (Fig 3). These cDNA panels represent pooled tissue samples obtained from different individuals, and allow accurate and precise gene expression analysis in a wide variety of tissues. The expression profile of the ST1C4 transcript resembled that known for A1AT, i.e. showed a high expression in the liver, lung, kidney, small intestine and leukocytes [28,29]. In contrast, the expression of ST1C5 transcripts was almost exclusively restricted to leukocytes. As illustrated in Fig 3, ST1C5 was detected in the lung tissue but expression levels accounted only for 20% of those seen in leukocytes. Although the commercial cDNA panels used for expression patterning of short transcripts provided a nice overview about their expression, it would be useful to perform a more detailed analysis in future studies on specific tissues and cell types.

**Fig 4:**
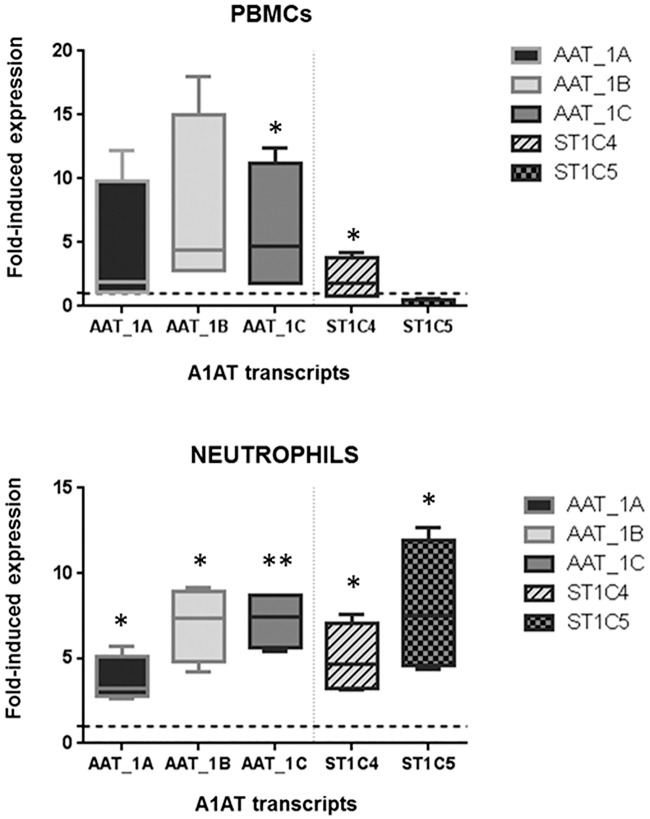
Induction of *SERPINA1* transcripts by LPS-treatment in isolated human blood PBMCs and neutrophils. Treatment of PBMCs with 100 ng LPS (n = 4 independent experiments) and neutrophils with 10ng LPS for 5 hours (n = 4 independent experiments) resulted in enhanced expression of full transcripts (1A, 1B and 1C) and short transcripts ST1C4 and ST1C5 relatively to the basal expression in untreated cells (represented by a dashed line). Box plots display variation in samples. Statistically significant values are shown by *(p<0.05) or **(p<0.01).

Since leukocytes showed the highest expression of both ST1C4 and ST1C5 transcripts, we next investigated the expression and regulation of these transcripts in human blood isolated PBMCs and neutrophils. Cytokines, chemokines, hormones and other substances control the constitutive and modulated expression, and release of A1AT [17,30]. These factors act specifically on different cell types and probably control the production of A1AT at sites of inflammation. Bacterial lipopolysaccharide (LPS), an inflammatory activator, modulates synthesis of A1AT in different cell types including neutrophils, monocytes and macrophages [31]. Therefore, we prepared freshly isolated human blood PBMCs and neutrophil cultures that were stimulated or not with LPS. As expected, LPS increased the expression of the full A1AT transcript in PBMCs and neutrophils (Fig 4). In PBMCs, LPS also induced the ST1C4 expression by 2 to 4-fold, but had no effects on ST1C5 expression. Neutrophils stimulated with LPS showed increased expression of both ST1C4 (by 2 to 8-fold) and ST1C5 (by 5 to 10-fold) if compared to non-treated neutrophils (Fig 4). In contrast, T lymphocytes did not show significant expression of either A1AT or short transcripts (data not shown).

### Detection of A1AT short C-terminal peptide in LPS-stimulated neutrophils

**Fig 5:**
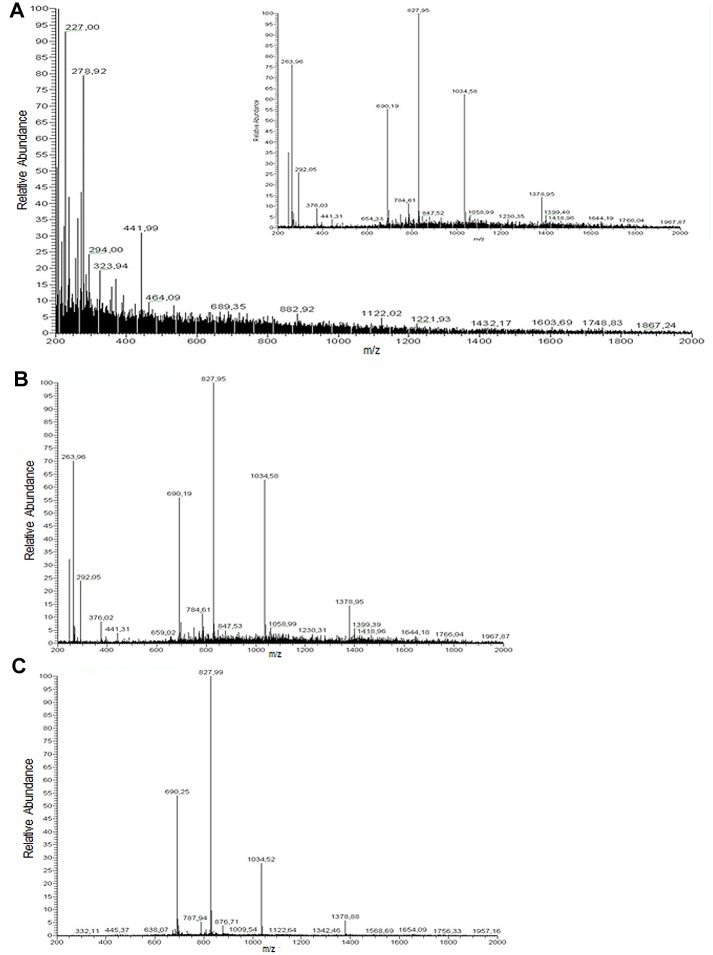
MS profiles of supernatants from cell cultures. A) shows the MS spectrum of a control sample (“blank”) and of the same sample spiked with an aliquot (1μM) of the standard C-36 fragment (inset). B and C) show to the profiles of LPS-stimulated cells for 2 h, obtained from two different experiments (indicated as LPS1 and LPS2). The multiple-charged ions ([M +H]6+; [M +H]5+; [M +H]4+; [M +H]3+), represent the fragment with 4134,9 ± 1 Da molecular size. For further details, see the text.

Since A1AT short transcripts ST1C4 and ST1C5 were highly induced in LPS-stimulated neutrophils, we speculated about the possibility to find A1AT peptides in cell culture supernatants. Therefore, we next performed LC-MS analysis in the cell culture supernatants from LPS-stimulated and not stimulated neutrophils. Fig 5A shows the MS profile obtained from non- stimulated cells (used as “controls”) and Fig 5B and 5C illustrate the profiles of LPS-stimulated cells (for 2 h), obtained from two independent experiments (indicated as LPS1 and LPS2). While non-stimulated cells (Fig 4A) do not show any significant signal, thus representing essentially a “blank”, LPS-stimulated cells (Fig 5B and 5C) contain clear signatures (at 690,25; 827,99; 1034,32 and 1378,88 m/z values) corresponding to the multiple-charged ion ([M +H]6+; [M +H]5+; [M +H]4+; [M +H]3+, respectively) with 4134,9 ± 1 Da molecular size. Using the Swissprot database, this fragment unambiguously was identified as a C-terminal fragment of A1AT (4135 Da). To “validate” this, in both experiments the real sample was spiked with an aliquot (1μM) of the standard, synthetic C-36 fragment. Not surprisingly, the pattern of m/z signatures was identical to that described above, the only difference being observed in the intensity of multiple-charged ions. Spiking the “blank” with 1 μM standard C-36 fragment resulted in the generation of a MS profile that was similar to that shown in Fig 5B and 5C. These latter results are presented in the inset of Fig 5A. The LC-MS runs performed on supernatants of cells that were LPS-stimulated for 5 h in two independent experiments gave essentially the same results to those illustrated in Fig 5 (data not shown). The results from the LC-MS experiments for the first time identify C-terminal peptide of A1AT in the supernatants of activated neutrophils but unfortunatelly do not answer the question of whether this peptide is generated after endogenous A1AT cleavage or is newly produced and secreted. Cleavage by target proteases (like neutrophil elastase or proteinase 3) triggers a conformational change of A1AT, in which the N-terminal portion of the reactive center loop spontaneously inserts into β-sheet A, carrying the bound protease (covalently linked as an acyl-enzyme intermediate) along with it. Cleaved C-terminal part of the A1AT typically remains bound to the larger part of the cleaved A1AT. However, under the specific conditions free C-terminal peptide of A1AT can be generated. For example, Frochaux V et al. [32] shows that the reaction of placental A1AT with HTRA1 leads not only to the formation of a protease-protease inhibitor complex, but also to the formation of cleavage fragments of A1AT. Therefore, future studies are needed to specifically address the question of whether the free C-terminal peptides of A1AT are newly synthesized.

So far, all described alternative human *SERPINA1* transcripts differ regarding their sites of transcription initiation and alternative splicing events, located in exons 1A, 1B and 1C at the 5’UTR region. Intriguingly, the new short transcripts variants are generated by alternative splicing affecting the coding exons, and all short transcripts include the last exon V of the gene. Exon V encodes a very important functional part of the A1AT protein, since it includes the active site (Met358-Ser359) as part of the reactive site loop of the A1AT, which acts as a docking site for binding and neutralizing neutrophil elastase [33]. Therefore, the 44 aa peptides are expected to contain the active site region and protease inhibitory activity. In support, the 44-residue C-terminal fragment of A1AT detected in placenta and human tissues [34], was found to inhibit neutrophil and pancreatic elastases [35].

A1AT is a well-known protease inhibitor forming stable complexes with a range of serine proteases, specifically neutrophil elastase and proteinase 3. A1AT can be targeted by non-specific proteases (e.g., matrix metalloproteases) without the formation of stable inhibitor complexes. (MEROPS database, http://merops.sanger.ac.uk/index.shtml). In both cases, interaction between A1AT and proteases results in cleavage of A1AT and irreversible loss of its inhibitory activity. Different studies provide evidence that native A1AT is proteolytically processed into firmly attached larger N-terminal and shorter C-terminal fragments [36]. Interestingly, in contrast to the biochemical and structural data, variants of A1AT C-fragments have been identified in human tissues and body fluids [34,37–41]. The physiological functions for these peptides have also been reported, like NK-cell suppression (CRISPP peptide)[42], extracellular matrix protection (SPAAT peptide) [43], pro- or anti-inflammatory immune modulating functions [41,44], and inhibition of HIV entry (VIRIP peptide) [40]. We previously reported that the C-terminal fragment of A1AT forms amyloid-like fibrils, activates human monocytes and is present in atherosclerotic plaques [45]. We also found C-terminal peptide of A1AT in the lungs of patients with chronic obstructive pulmonary diseases. [41]. Recent studies revealed that so called cell-penetrating peptides (CPPs) have the ability to cross cellular membranes, either alone or in association with bioactive cargo. The C-terminal peptide of A1AT, 105Y (CSIPPEVKFNKPFVYLI), has been characterized as endoporter for siRNA [46]. Hence, the effects of C-terminal peptides in vivo are likely to be complex. It remains to be confirmed if free C-terminal peptide(s) of A1AT are exclusively produced from the A1AT cleavage or/and can be *de novo* synthesized and released by different cells.

In summary, we identified novel short transcripts of the SERPINA1 gene, differentially expressed in tissues and cells. We also provide experimental evidence that the expression of short transcripts can be regulated and that C-terminal peptides of A1AT can be found in free form in LPS stimulated neutrophils.
